# Whole-Body Magnetic Resonance Tomography and Whole-Body Computed Tomography in Pediatric Polytrauma Diagnostics—A Retrospective Long-Term Two-Center Study

**DOI:** 10.3390/diagnostics13071218

**Published:** 2023-03-23

**Authors:** Marnie Raimann, Johanna Ludwig, Peter Heumann, Ulrike Rechenberg, Leonie Goelz, Sven Mutze, Vera Schellerer, Axel Ekkernkamp, Mustafa Sinan Bakir

**Affiliations:** 1 Center of Orthopedics, Trauma Surgery and Rehabilitative Medicine, University Medicine Greifswald, 17489 Greifswald, Germany; 2 Department of Trauma and Orthopedic Surgery, BG Klinikum Unfallkrankenhaus Berlin gGmbH, 12683 Berlin, Germany; 3 Kellogg College, University Oxford, Oxford OX2 6PN, UK; 4 Department of Radiology and Neuroradiology, BG Klinikum Unfallkrankenhaus Berlin gGmbH, 12683 Berlin, Germany; 5 Institute for Diagnostic Radiology, University Medicine Greifswald, 17489 Greifswald, Germany; 6 Department of Paediatric Surgery, University Medicine Greifswald, 17489 Greifswald, Germany

**Keywords:** children, MRI, CT, trauma, diagnostics

## Abstract

Although serious accidents remain the leading cause of pediatric mortality, protocols to orient diagnostic procedures towards a certain type of initial imaging are widely needed. Since 2007, we have performed whole-body magnetic resonance imaging (WBMR) and whole-body computed tomography (WBCT) for diagnoses of severely injured children. We retrospectively reviewed 134 WBMR and 158 WBCT in patients younger than 16 years that were performed at two trauma centers between 2007 and 2018. A higher Injury Severity Score (ISS) was found in WBCT vs. WBMR (10.6 vs. 5.8; *p* = 0.001), but without any significant difference in mortality. The WBMR was significantly preferred at younger ages (9.6 vs. 12.8 years; *p* < 0.001). The time between patient’s arrival until diagnosis was 2.5 times longer for WBCT (92.1 vs. 37.1 min; *p* < 0.001). More patients in the CT group received analgesic sedation and/or intubation at 37.3% vs. 21.6% in the MRI group. Of these patients, 86.4% (CT) and 27.6% (MRI) were already preclinically sedated (*p* < 0.001). Correspondingly, 72.4% of the patients were first sedated in-hospital for MRIs. In conclusion, WBMR is an alternative and radiation-free imaging method for high-energy-traumatized children. Although the selected diagnostics seemed appropriate, limitations regarding longer duration or additional analgesic sedation are present, and further studies are needed.

## 1. Introduction

In industrialized countries, accidents are still the main cause of mortality in children [1,2,3,4]. Teenagers (62.0%) represent the largest fraction of this group, and in the age range of 1 to 4 years, 19.8% are affected by a polytrauma that leads to death [2]. Whole-body computed tomography (WBCT) is generally recommended in adult patients with suspected polytrauma. In Germany, WBCT is indicated according to the guidelines of the German Society of Traumatology (DGU) [2]. Its superiority concerning specificity and sensitivity in detecting solid organ injuries and the considerable reduction in time to diagnosis, when compared with conventional radiography or selective CT imaging, are seen as a notable advantage in polytrauma management and also in terms of increased survival [5,6,7,8].

Dedicated guidelines as to how to proceed with polytraumatized children have been vague and not standardized for a long time [3]. A German S2k guideline was published in 2021, but it was based on minor evidence and produced recommendations based on structured expert consent [9]. However, quick and thorough imaging of the whole body via WBCT entails radiation risks, such as an increased incidence of scan-related cancer, especially with decreasing patient age [10,11,12,13]. Thus, in the course of rising awareness in reducing radiation exposure, implementation of whole-body magnetic resonance tomography (WBMR) as an alternative for CT for primary imaging, after pediatric high-energy trauma, appears logical [14]. Comparable to body-imaging with WBCT, WBMR can be used to detect bone lesions and hemorrhage in addition to injuries of inner organs. However, a certain latency to diagnosis, due to longer scan times and a possible need of anesthesia or sedation, have to be accepted and a limited access to the imaging modality of WBMR, at any time, can certainly not be ensured for every hospital at the moment [15,16]. As previously shown, WBMR use seems to be advantageous, especially in conscious and hemodynamically stable pediatric patients after high-energy trauma [17]. However, neither the benefits of using WBMR, nor a decrease in mortality by using WBCT, in case of polytraumatized children, have been proven evidentially yet [2,17,18]. Therefore, heterogeneous management is seen in clinical practice [18]. Standardized working processes, containing evidence-based recommendation for diagnostic workups, are desirable because they would allow physicians to use these algorithms without delay and help save precious time in emergency cases [19].

Further research is needed concerning this less investigated topic of appropriate radiological imaging selection in case of high-energy trauma in pediatrics [18]. Therefore, this retrospective observational study analyzes whether and in which cases WBMR could serve as a radiation-free alternative to WBCT for imaging in the pediatric population after polytrauma, despite longer examination times. We aim to analyze the frequency (or rather the dependency) of WBCT and WBMR in relation to certain variables and to develop evaluative statements as to how these techniques were used in our study population, with the objective of detecting broadly valid patterns. The primary hypothesis is that, in the subgroup of conscious and hemodynamically stable children, the preferential use of WBMR instead of WBCT is an equivalent method for detecting moderate injury severity, as described by the Injury Severity Score and Abbreviated Injury Scale (ISS/AIS). As a secondary objective, it is supposed that the following parameters influence the choice of imaging method: (1) age, (2) expected urgency, and (3) trauma mechanism. In addition, outcome relevant factors, such as the time from arrival until primary imaging, additional diagnostics, injury-patterns, dwell-time, and (surgical) interventions, were evaluated.

This retrospective study serves to show that WBMR could be an alternative to WBCT in high-energy traumatized children, and that WBMR is equally qualified in terms of predicting mortality.

## 2. Materials and Methods

**Figure 1:**
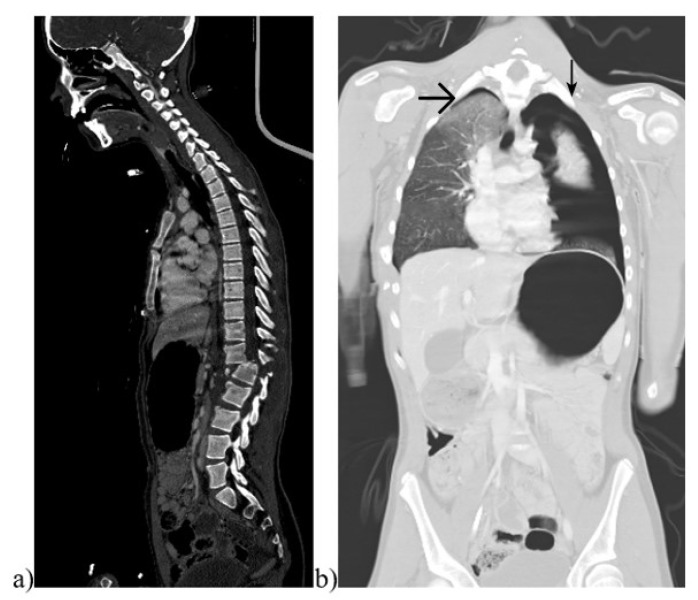
Images of a pediatric whole-body computed tomography (WBCT). Example of a 14-year-old girl with a traffic accident as a passenger in a car. CT images showing (**a**) a luxation fracture T12/L1 on sagittal plain in a bone window and (**b**) a pulmonary contusion and bilateral pneumothoraces on coronal plain in a lung window (marked by arrows).

This retrospective study included pediatric patients who were hospitalized between 2011 and 2018 at two level 1 trauma centers and had undergone WBCT or WBMR after suspected polytrauma (Figure 1). Patients up to the age of 16 years were enrolled if a clinical evaluation did not seem sufficient and there was a common decision for whole-body diagnostics.

Diagnostic findings, radiology reports, discharge letters, emergency room documentations, nursing reports, and coding information served as sources of information. All primary data, such as radiological reports, were checked at the senior consultant level and were extracted/supervised by at least two experienced experts.

**Figure 2:**
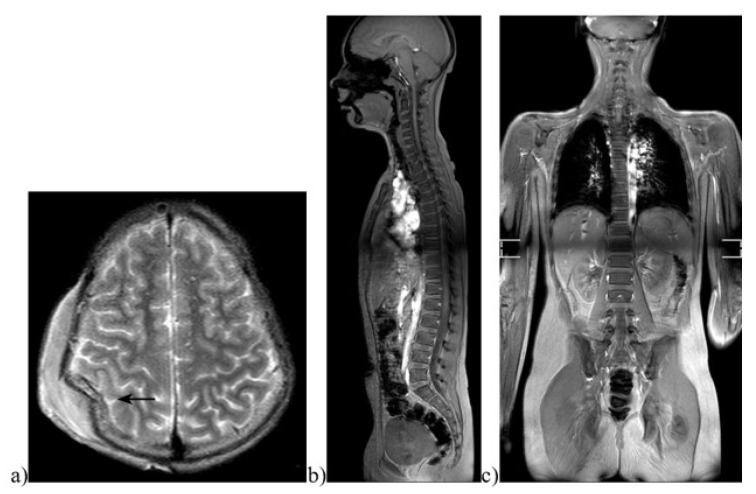
Images of a pediatric whole-body magnetic resonance tomography (WBMR). Example of a 12-year-old boy who was hit by a car as a pedestrian. MRI images showing (**a**) a large compression fracture and subgaleal hemorrhage on axial TSE T2w imaging (marked by an arrow). The (**b**) sagittal STIR and (**c**) coronal STIR full-body images showed no spinal fracture, parenchymal lacerations, or free fluid indicating further injuries. A standard protocol developed specifically for pediatric whole-body imaging via MRI was used [17].

WBMR was only considered as an alternative diagnostic method for conscious and hemodynamically stable pediatric patients and was initiated according to the trauma team’s decision, without further criteria (Figure 2). A cardiovascular affection requiring stabilization prior to imaging was an exclusion criterion for WBMR, and a WBCT was performed. A recently published WBMR standard examination protocol was used, including MR imaging from the apex of the head to the pelvis [17]. For WBCT, the standard protocol consisted of the following: (1) non-contrast spiral-head CT: 120 kv, modulated mAs with dose reduction software Care Dose, Siemens, Akq. 64 × 0.6 mm; (2) spiral scull base to os ischium: (down)modulated mAs and kv from 100 mAs and 120 kv, Akq. 128 × 0.6 mm, Imeron 300 in split-bolus technique total volume 2 mL/kg BW −10 mL and same volume of NaCl flush.

As variables, age, sex, date of admission, and discharge of the patients, including the time from arrival until first imaging, were evaluated. Furthermore, preclinical data, such as the mode of arrival at the hospital by a rescue helicopter or by ambulance, the triage category according to the Manchester Triage System, and the need of treatment in the resuscitation room/emergency department, were recorded [20]. Classification in the red triage category means that immediate medical treatment is required; indicators for this selection were compromised airways or inadequate breathing, life-threatening bleeding, unresponsive children, and shock.

The causes for trauma were listed in detail and were categorized into severe and mild trauma mechanisms. Severe trauma mechanisms included traffic accidents as pedestrians, as cyclist vs. car, any motorized accidents, and falls from a height ≥ 3 m. Accidents while driving on a bicycle or a scooter, falls from heights < 3 m, and all other detected causes were classified as mild trauma mechanisms. Among mild trauma mechanisms, “others” included explosive device injuries, physical injuries caused by violence, and rollover traumata in a buggy or sled, in addition to other extraordinary events. Non-traumatic indications for whole-body imaging were excluded from the analysis.

In cases of intubation or analog-sedation, whether these children had been intubated or sedated preclinically, or whether this procedure was necessary to perform diagnostics, was analyzed.

The need for additional imaging before or after whole-body imaging was analyzed with regards to CT, MRI, WBMR, WBCT, and radiographs of individual body regions. As a standard in emergency management, Focused Assessment with Sonography for Trauma (FAST) and additional sonographic follow-up examinations were excluded from analysis. To prevent bias in interpretation of MRI and CT, results of additional control imaging, which were not strictly part of the emergency protocol, have been excluded from analysis.

According to the outcome data and the international Abbreviated Injury Scale (AIS), the injuries were subdivided into the following body regions: head, face, thorax, abdomen, spine, upper extremity, lower extremity, and vessels [21]. Injuries that affected the vessels were analyzed separately. Subsequently, the Injury Severity Score (ISS) was determined for all patients based on the AIS. The number of affected body regions was also recorded in the cohorts, with a separate subgroup analysis of AIS ≤ 2 and AIS ≥ 3.

The proportion of patients who had undergone surgeries or interventions was noted, in addition to the number of days in the peripheral ward or in an intensive care unit (ICU). Surgeries and interventions included the placement of thoracic drainages, but excluded small skin lacerations of the face and head that could be treated by tissue glue or by stitches in the emergency room.

In case of variables of two independent random samples with absolute qualitative characteristics, and when *n* was ≥5, statistical analysis was performed with chi-squared test by Pearson, while in cases of *n* ˂ 5, chi-squared test by Fisher’s exact test was performed. Significance was assumed at an alpha of *p* ≤ 0.05. A student’s *t*-test was used in case of metric measurements. Results were presented as mean with standard deviation (SD) and percentages in case of categorical data. A 95% confidence interval (95% CI) was calculated for selective rates. SPSS statistical software was used for analysis (Version 26, IBM Inc., Armonk, NY, USA).

The study was registered with the German Clinical Trials Register (DRKS; DRKS00017015) and was approved by the local ethics committee (University Medicine Greifswald, BB 016/19; date of approval: 1 March 2019).

## 3. Results

Two patients in the WBCT group were excluded because of cardio-respiratory failure without evidence for trauma. Another patient in the WBMR group was excluded because the symptoms arose without the presence of current trauma. In total, data from 290 patients were evaluated. The WBMR group contained 134 and the WBCT group contained 156 patients.

### 3.1. Descriptive Parameters

The average Computed Tomography Dose Index (CTDI) in the CT group was 6.1 mGy (±3.5 mGy) and the Dose-Length-Product was 581.8 mGycm (±418.8 mGycm). The average amount of contrast medium was 118.5 mL (±37.4 mL). Four different CT scanners were used (Philips Ingenuity TF, Philips Brilliance CT Big Bore, Philips Diamond Select Brilliance 16 CT; Amsterdam, The Netherlands; Siemens SOMATOM Force; Munich, Germany). The duration of the scan and the preparation lasted 4–7 min, while it lasted 15–20 min in the MR group. Three different MRI scanners were used (Philips Panorama HFO 1 Tesla, Philips Intera 1.5 Tesla, Philips Achieva 3 Tesla; Amsterdam, The Netherlands).

As an imaging method, WBMR was used significantly more often in patients of younger ages, especially below the age of 13 years (Table 1). The sex of the patients did not differ significantly in both body imaging groups.

### 3.2. Preclinical Data

As a marker for the preclinical estimated severity of the trauma, twice as many of the patients that arrived via rescue helicopter were diagnosed via WBCT than by WBMR (Table 2). A similar, significant difference existed for the group of red triaged patients who were mostly examined via WBCT. However, the level of triage was only documented in 2/5 cases of the WBCT group, and triage data in the WBMR group was missing in another four cases.

In terms of trauma mechanisms, severe trauma, especially a motorized accident or a fall from a height ≥ 3 m, led significantly more often to the use of WBCT (Table 3). In contrast, children who were involved in an accident as pedestrians, or trauma mechanisms that were categorized as minor falls, were more often diagnosed using WBMR (Table 3).

### 3.3. Clinical Data

Patients examined via WBCT went, significantly more often, directly into the resuscitation room/ED after arrival at the hospital (87.8%; *p* < 0.001). In contrast, 67.9% of the patients, in which WBMR was chosen as the imaging method, were first seen in the resuscitation room. The time period between the patients’ arrivals until WBMR (92.2 ± 118.4 min) was significantly longer (*p* < 0.001) than the mean time periods from arrival until the WBCT scan (37.1 ± 39.2 min).

Overall, in the course of major trauma treatment, analog-sedation or intubation was necessary significantly more often in the WBCT group, especially in those who had already been treated preclinically.

In-hospital intubation was observed frequently in the WBMR group and occurred almost four times more often than patients who underwent WBCT scans during the emergency room phase.

In the WBMR group, intubation or analog-sedation were used for diagnostic reasons in 8 of the 134 patients (5.9%). These procedures were less frequent in the WBCT group with two cases (1.2%). One of these patients underwent additional radiography of the thorax and the other one underwent a cranial MRI (Table 4).

In children who underwent WBMR, more additional imaging was observed. In particular, a significantly increased number, prior to whole-body diagnostics, is shown for the MR group compared to the CT group (Table 5).

### 3.4. Outcome Data

A significantly higher ISS was found in WBCT (10.6 ± 12.1) in contrast to the WBMR group (5.8 ± 4.3; *p* = 0.001). The range of ISS showed its median at 6 in the WBCT group (interquartile range Q1/Q3: 4/13), while the median was 5 in the WBMR group (interquartile range Q1/Q3: 3/8). Significantly more children got an ISS ≥16 after WBCT (*n* = 33, 21.2%; *p* < 0.001) in contrast to the WBMR group (*n* = 4, 3.0%). On average, patients in both groups sustained injuries of two body regions without significant differences. In the WBCT group, significantly more injuries affecting the facial area and the thorax were detected. Abdominal, spinal, and lower extremity traumata were found significantly more often in the WBMR group (Table 6).

In the WBMR group, the resulting AIS of almost all body regions was <2, while in the WBCT group, AIS was significantly higher, with an average AIS > 2, with the exception of cranial injuries (Table 7).

In the group of patients with AIS ≥ 3, children in the age range of 13 to 16 years were mainly and significantly examined more often via WBCT, whereas MRI was only used in 1.4% of cases in this age-category (Table 8).

Patients with AIS ≥ 3 were examined significantly more often with WBCT when injuries of the thorax, abdomen, and lower extremities occurred. Additionally, based on this subanalysis, the portion of severe injuries was higher in the CT group.

After severe trauma, a significantly higher injury severity was found in the WBCT group (Table 9). In general, a higher ISS was found in the WBCT group (9.5 ± 7.7 and 7.2 ± 5.8) than in the WBMR group (6.4 ± 4.7 and 5.0 ± 3.3).

Patients who underwent WBCT were in significant need of surgery or another intervention more often, such as a thoracic drainage, in contrast to the WBMR group (CT 49.3% vs. MRI 18.6%, *p* < 0.001).

Concerning hospitalization, patients who were examined via WBCT stayed at the hospital more than three times longer and stayed in the ICU nine times longer than the WBMR group (Table 10). No significant difference in mortality was found between groups; two patients in the WBCT group died during the study period, while no one deceased in the MR group.

## 4. Discussion

In our study, WBMR appears to be an advantageous tool, with regards to ionizing radiation exposure, and a suitable alternative for pediatric high-energy trauma diagnostics. The decision making between CT and MRI, based on appropriate patient selection, as presented, seems to be adequately performed, since severely injured children still received a WBCT and mildly injured children were diagnosed via WBMR [17].

However, the severity code of injuries is confronted with the radiation exposure of several examination methods. As the fastest imaging method, WBCT provides findings in a few seconds, but a huge disadvantage is the extensive radiational burden from ∼2.5 mSv in neonates up to 29.5 mSv/adult weight patient because the organs of children are more susceptible to radiation, and the incidence of scan-related cancer increases with the decreasing age of the patient [11,12,13,22]. In female children, an additional lifelong risk of breast and lung cancer seems evident [11]. However, evidence-based predictions about concrete risks of WBCT-scan-induced cancer are unfeasible at the moment, since preventing unnecessary CT scans has proven to be successful in minimizing cancer risks [2,7,11,18,23]. Although the principle of radiology, “as low as reasonably achievable” (ALARA), is generally accepted, literature describes cases in which CT is still used for primary diagnosis, without the need for consecutive intervention [24]. Therefore, the major advantage of the WBMR is to prevent radiation exposure in contrast to WBCT, resulting in eliminating the radiation risks of scan-related cancer, particularly highlighted in the youngest age groups [10,11,12,13,14]. For choosing an imaging method, several studies confirm that the age of a child should be more frequently considered, in addition to the child’s state of consciousness and the clinical findings, instead of merely relying on mechanisms of injury [2,10,24]. The excellent soft-tissue contrast provided through WBMR, and the additional huge advantage of an absence of ionizing radiation, makes WBMR an attractive imaging modality for children, especially in cases of repeated follow-up examinations [16,25].

The decision making for a respective diagnostic method is based on preclinical facts and the primary clinical presentation in the emergency room, which seems to lead to an appropriate assignment to either WBCT or WBMR. The preclinical variables for decision making are those that are available prior to diagnosis. What seems conclusive at first glance should be reviewed again since the whole extension and severity of an injury can certainly only be revealed after imaging [7,18]. Facts and data prior to imaging, which indicate a greater injury severity, such as arrival by helicopter, a high triage category, or the direct admission to the resuscitation room after committal, lead to the indication of a WBCT as diagnostic modality. Yeguiayan et al., who analyzed diagnostic imaging with CT and WBCT, already described that the trauma mechanism, especially, and the initial medical treatment seem to influence diagnostic approach [5,26]. Further clinical data, such as hemodynamic parameters (e.g., heart rate, blood pressure, breathing rate), laboratory tests (e.g., hemoglobin, lactate), and pediatric Glasgow Coma Scale, have an influence on the choice of the imaging method, too. Although a hemodynamic affection requiring stabilization prior to imaging was an exclusion criterion for WBMR, according to our house-standard algorithm, an analysis of these influencing factors was not part of this study. However, it cannot be decided with certainty whether the selection of an imaging modality was indeed based on the expectation of more severe trauma, due to the retrospective nature of this study, since this design does not allow any causal statement. Nevertheless, we conclude that our results underline our previous research and that our decision criteria for the preferential use of WBMR as a radiation-free alternative imaging method for non-life-threatening injured and conscious pediatric patients is feasible [17].

In this context, it should be underlined that, despite the clinical decision on a child’s sufficient stability for the more time-consuming imaging method WBMR, the interdisciplinary team of trauma surgeons and radiologists limited the extent of the examination protocols as much as possible, with a focus on the imaging duration. While other MRI techniques, like metabolic and dynamic imaging, have the potential to revolutionize insights into the molecular level of trauma, their applicability in acute diagnostics and treatment, especially in children, are far beyond current comprehension [27]. MRI protocols also excluded administration of Gadolinium, which could have been used for imaging of the great vessels because non-contrast techniques, like steady-state free precession imaging, have shown similar results compared to contrast-enhanced imaging [28]. Nevertheless, dedicated vascular imaging was never part of the standard WBMR protocol because we aimed to exclude children at risk of great vessel injuries from WBMR through rigorous evaluation of preclinical and clinical information. This defensive approach and the lack of knowledge about the sensitivity of MRI, i.e., for vascular injuries in children after severe trauma, could result in an underdiagnosis of certain injuries and, as a consequence, in adding up to an uncertainty about the injury severity of children diagnosed using WMBR.

Motorized accidents in road traffic are described as the main trauma cause in 80 to 90% of severe injuries in polytraumatized children [3,14]. This finding was confirmed in our observation, resulting in a high Injury Severity Score after these severe trauma mechanisms. In our observations, children of all age categories who were injured through severe trauma mechanisms showed a higher injury potential that led to examination via WBCT significantly more often than WBMR. Higher Injury Severity Scores were found among teenagers. We speculate that teenagers participate independently in road traffic and might not be as cautious and as well spotted as adults. In addition, the usage of scooters or mopeds should not be underestimated in this age range [26]. This finding is in line with previous research which found that children < 6 years suffer accidents more often as pedestrians, car passengers, or due to falls from great heights [14,26]. Nau et al. described that children at the ages of 5 to 8 are beginning to act independently and are therefore more endangered, while not being able to comprehend dangerous situations [3,14]. School-aged children, from seven to twelve years old, start to undertake activities within traffic areas and are more affected by accidents as pedestrians or bicyclists. Teenagers, up to 17 years, were found to be involved in car, bicycle, or moped accidents [26].

After trauma mechanisms, which seemed to indicate milder trauma, patients more commonly underwent WBMR. Pedestrians in traffic accidents underwent WBMR significantly more often than WBCT. That finding might show a tendency similar to results of recent studies in children, revealing that mortality is lower after injury as pedestrians when compared to adults [26]. Lower-energy trauma, when pedestrians are hit by a rather slow vehicle, could be an explanation for milder trauma and the choice of WBMR. In contrast, higher ISS of pedestrians were seen in our WBCT group, a finding which supports the hypothesis that severely injured children were preferentially examined via WBCT.

Preclinical intubation also seems to be assumed as a predictor of severe trauma in children [18]. The undeterminable neurological status in preclinically intubated patients probably co-factored the indication for WBCT in these cases [10]. An expected high injury severity, later confirmed by high ISS, obviously indicates preferable use of WBCT. Therefore, a higher number of intubations in the WBCT group, due to the higher ISS, was noted. On the other hand, clinical intubations, in addition to solitary analog-sedation, especially those for diagnostic reasons, were seen more often in the WBMR group. This finding appears logical when taking into consideration the necessity of sedation for the longer examination period needed for WBMR. Additionally, WBMR was mainly used in younger patients who were examined without radiation but could not lie calmly during imaging.

In the latter cases, a longer period of time before diagnosis in the WBMR group might be acceptable because of less urgency during diagnostics. This is an important aspect since the time period until WBMR was more than twice as long from arrival until WBCT, and the execution of WBMR lasted longer, too. Arriving patients with red triage, who seem to be more severely injured, are generally examined as quickly as possible. Apparently, the shorter time to diagnostic findings resulted in the choice to perform WBCT more often. One should keep in mind that an arrival of multiple traffic accident-injured patients in the same time period could possibly delay diagnostics, due to the occupancy of the required equipment, and could also lead to a deferral of the particular examination. In addition, the number of available CT and MR scanners differs among clinics. During implementation of WBMR, 24/7 availability could not be ensured, but after standardization, WBMR was available all around the clock in our institution. However, it must be acknowledged that the 24/7 availability of WBMR cannot be assured in every hospital. In these cases, alternative diagnostics of children using an ultrasound, followed by a clinical (intensive care) observation are options which were not part of the current analysis. The group of hemodynamically stable, conscious pediatric patients should be evaluated with an ultrasound plus observation vs. WBMR in further prospective research. As another potential alternative to (abdominal) imaging, some studies tried to implement special prediction rules using physical examination and laboratory testing after blunt trauma in children, which could help contribute to the identification of intra-abdominal injuries, and decide whether CT imaging is necessary, or otherwise reduce radiation exposure [29,30].

When analyzing additional diagnostics, depending on the trauma mechanism, it was notable that in pedestrians, especially, who were initially examined via WBMR, additional imaging was added. A possible cause could be that children injured in road traffic accidents suffered more often from bony fractures, which are usually better identifiable on radiographs than MRI. WBMR is performed down to the level of the pelvis, so fractures of the lower extremity are not detected. Radiographs were essential complementary exams for both patient groups, since they produce a lower radiation dose, and were better than expanding the WBCT to the lower extremities, which is even more important in childhood.

Furthermore, the higher number of additional imaging before WBMR than before WBCT might be related to the longer time to diagnostic results and the faster availability of CT imaging, even in well-staffed institutions. Several radiographs of injured body regions/extremities might be acquired while waiting until the MRI is ready for use. The same could also apply to immediate CT imaging of head and cervical spine, which, in some cases, may have already been performed prior to the WBMR in order to avoid a delay of a related time-critical intervention. Although no significant differences between the groups concerning the total number of additional imaging after WBCT or WBMR was seen, it is interesting to note that supplementary examinations were required in nearly equal parts.

Current literature mainly focuses on comparisons between WBCT and focused CT scanning as diagnostic methods in emergency cases [7]. Therefore, literature concerning WBMR is limited. Based on the determined AIS and ISS, it was shown that, on average, patients examined via WBCT were more severely injured than the patients examined via WBMR. Apparently, severe single injuries (AIS > 2) that were detected in almost every body region, other than the upper extremities and the facial regions, provided a reason for preferential examination via WBCT instead of WBMR. The significantly more severe injuries of every body region in the CT group leads to the conclusion that the prudent choice between CT and MRI was made correctly, and enabled the diagnosis of severe injuries with the quicker WBCT.

Literature specifies that injuries of the same body regions occur most frequently in all age categories and cause the highest mortality [10,26,31,32]. Taken together, the injury pattern was fairly analogous to previously published data [10,19,26,31]. In our study, injury patterns, in general, did not indicate more injuries of the head in the WBCT than in the WBMR group, but the more severe cases of head trauma were found in the WBCT group. That finding could be explained by the statement of the German Society of Traumatology, which lists cranial CT as a facultative method and recommends MRI due to a higher sensitivity and specificity for parenchymatous injuries [2]. Analogous to Auner et al. for abdominal imaging, Kuppermann et al. developed prediction rules for imaging choices after head injuries. On the basis of clinical findings, the likelihood of mild head injuries was analyzed to avoid unnecessary CT scans [33]. It can be discussed that this approach could be refined using WBMR as an adjunct method to detect head injuries, as part of daily trauma management, without additional radiation in contrast to the standard currently dominated by WBCT.

Higher ISS values were found in children after severe trauma mechanisms. AIS and ISS evaluation, in cases of severe injuries of the head, thorax, and abdomen, and immediate detection of the extend of injuries via timesaving methods, have high priority in order to decrease mortality [2,18,26]. This approach was successfully performed in our study, even though WBMR was available at all times.

Moreover, in patients who were expected to require a surgery/intervention, WBCT was chosen, in most cases, as the quickest and best timesaving method for whole-body imaging. Walz et al. elaborated that persistent hemodynamic instability of a child leads to surgical interventions, rather than findings of free fluid [14]. The time to an eventual surgery/intervention cannot only be evaluated by deductive reasoning from the time from arrival to imaging because other determining factors are left to consider, such as capacity of CT/MRI scanner, emergency room, and operation room, due to other emergent cases treated at the same time.

Remmers et al. mentioned that polytraumatized children spend less time in hospital wards than adults [26]. We showed that after WBCT, patients had a longer length of stay both in peripheral wards and in the ICU. Higher severities of injury can be concluded retrospectively, accounting for the longer hospitalization in total. However, both extended length of stay in the peripheral ward and in the ICU, in the WBCT group, again serve as indications that the choice of imaging was exercised with caution.

A key limitation is certainly the missing standard operation procedure (SOP) for the use and choice between WBMR and WBCT. Although WBMR was on option only in cases of cardiopulmonary stability, no distinct cut-off values or exclusion criteria existed. Therefore, the decision of the diagnostic method still underwent subjective influence of the responsible trauma team in charge.

The two level 1 trauma centers implemented the triage from a specific date during our evaluation period, and thus, triage results were not documented in all cases. Nevertheless, if we assume a constant distribution of injury severity over the years, it can be concluded that the triage levels were also distributed in the same way. Among spinal injuries, which were detected 10% more often in the WBMR than in the WBCT group, clinical irrelevant bone bruises were taken into account and increased the number of spinal injuries in this group. This entity is not detectable on WBCT but has no further impact on treatment strategy. Because of this, further parameters, such as ISS, could have been influenced, although the single AIS impact was small. On the contrary, since WBMR has no diagnostic gold standard yet and its quality needs to be proved, there is also the risk that injuries might be overlooked, resulting in an incorrectly minor ISS. On WBMR, we also detected minor amounts of abdominal free fluid in some cases, which was later deemed physiological in these children. Those two findings are sensitively detected using WBMR but should neither draw attention away from relevant injuries nor initiate overtreatment.

Another potential bias results from the children who died a few days after initial treatment. These patients had a short hospitalization period, not despite, but because of the severity of their injuries. As only two such cases are found in our cohort, we conclude this factor to be rather negligible for our results. Additionally, one of the participating trauma centers does not provide a dedicated ICU for small children; children were preferentially transferred into specialized units and thus, the fraction of monitored children requiring ICU treatment could be biased. Hence, these cases have been excluded from analysis, but patients with an early transfer before inpatient admission to the center might have been missed. Furthermore, surgeries during the following days after trauma, which could not be performed directly after trauma due to unstable vital signs, are not listed as primary treatment and could not be differentiated in this retrospective study. In the group of patients who were already intubated or sedated upon arrival at the ER, it remains unclear whether they would have needed those procedures for diagnostic reasons.

The development of a SOP at the participating centers for the use of whole-body diagnostics in severely injured children, based on our results, was a consequence of our study. It should be focused on defining clear indications concerning choice of diagnostic methods after clinical assessment of (possibly) polytraumatized children [7,18]. Naturally, the evaluation of this SOP is the next necessary step, and further research concerning WBMR as an alternative diagnostic tool is crucial in the future. It must be emphasized that this study was focused on factors, such as the mechanisms of accidents, the preclinical course of traumatized children, and clinical parameters, to decide on the required imaging modality. However, there are biomarkers, such as interleukin-6 and troponin, which correlate with the injury severity after trauma in children [34,35]. Currently, it remains uncertain if these molecules are significantly elevated as early as minutes or a few hours after trauma. Future research might show if biomarkers could be another factor in guiding imaging decisions in severely injured children. The aim of this current study was indeed to highlight a proof of concept of a well-established technique, WBMR, in the new target group of severely traumatized pediatric patients. 

It remains difficult to choose the appropriate imaging modality based on conjecture, external physical signs, putative instability, and concomitant factors, such as the trauma mechanism. The detection of severely injured patients and adjustment of the needed resources for treatment is still an interesting issue of ongoing research in adults, but, of course, also in pediatric patients [36,37]. There is no evidence for using WBMR in the case of polytraumatized children with unstable circulation, in our opinion. Furthermore, as there are obvious advantages of implementing WBMR into primary diagnostic guidelines in polytraumatized children, innovative WBMR technologies could optimize protocols concerning shorter examination time, while remaining high image-resolution and future supplementary use of molecular markers could improve structural imaging in the assessment of internal damage [14,27,37]. However, research concerning this topic currently appears sparse and warrants priority. Future observations and, especially, prospective studies are vital to approximate evidence-based guidelines for the management of severely injured children and to verify the results of this retrospective study on a large scale.

## 5. Conclusions

The study at hand shows that several preclinical parameters indicate severe trauma mechanisms or unstable vital signs in children, and trigger examination via WBCT as the current imaging method of choice. In cases of suspected grave hemorrhage and/or violent severe trauma, and if urgent surgery appears necessary, WBCT was chosen as the most timesaving diagnostic-method. WBMR was performed more often in moderate trauma mechanism as the preferred diagnostic-method. Younger children benefit from examination via WBMR if they are expectedly moderately injured (AIS ≤ 2), since it is a radiation-free alternative, which is an important advantage for the most vulnerable youngsters. However, WBCT is currently not replaceable in severe cases because of the longer examination times and accessibility in many trauma centers.

## Figures and Tables

**Table 1:** Average ages in both imaging groups (±standard deviation) and percentages (and total numbers) in different age categories and sex distribution. All ages are presented in years. * = statistically significant difference.

	WBCT	WBMR	*p*-Value
Mean Age	12.8 (±3.8)	9.6 (±3.7)	<0.001 *
0–6	9.6% (15)	22.4% (30)	0.003 *
7–12	24.4% (38)	56.7% (76)	<0.001 *
13–16	66.0% (10)	20.9% (28)	<0.001 *
Male/Female	64.7%/35.3% (101)/(55)	64.2%/35.8% (86)/(48)	0.920

**Table 2:** Arrival via rescue helicopter and portion of red triaged patients in both groups. Values are given in percentages (and total numbers). * = statistically significant difference.

	WBCT	WBMR	*p*-Value
Arrival via rescue helicopter	27.5% (43)	13.4% (18)	0.003 *
Red triage	33.7% (53)	4.5% (6)	<0.001 *

**Table 3:** Severe and mild trauma mechanisms. All values are given as percentages (and total numbers) of all patients corresponding to each group. RTA: road traffic accident. * = statistically significant difference.

	WBCT	WBMR	*p*-Value
Severe trauma mechanism	80.8% (126)	61.9% (83)	<0.001 *
Pedestrian RTA	19.2% (30)	29.1% (39)	0.049 *
RTA motorized	32.0% (50)	12.6% (17)	<0.001 *
RTA bicycle vs. car	13.4% (21)	13.4% (18)	0.994
Fall ≥ 3 m	16.0% (25)	6.7% (9)	0.014 *
Minor trauma mechanism	19.2% (30)	38.1% (51)	<0.001*
Fall < 3 m	5.7% (9)	26.8% (36)	<0.001 *
Bicycle/Scooter	3.2% (5)	4.4% (6)	0.572
Others	10.2% (16)	6.7% (9)	0.284

**Table 4:** Intubation (IT)/analog-sedation (AS). All values are given in percentages (and total numbers). * = statistically significant difference.

	WBCT	WBMR	*p*-Value
All patients with IT/AS in total	36.5% (57)	21.6% (29)	0.006 *
Preclinical IT/AS related to all Patients	31.4% (49)	6.0% (8)	<0.001*
Preclinical IT/AS related to IT/AS in total of each imaging group	85.9% (134)	27.5% (37)	<0.001 *
IT/AS for diagnostic reasons	1.2% (2)	5.9% (8)	0.048 *

**Table 5:** Additional diagnostic imaging including radiographs and selective CT/MRI. All values are presented in percentages (and total numbers). * = statistically significant difference.

	WBCT	WBMR	*p*-Value
Imaging before WBCT/-MR	8.3% (13)	24.6% (33)	<0.001 *
Imaging after WBCT/-MR	64.1% (100)	60.4% (81)	0.522

**Table 6:** Affected body regions in both imaging methods. Severe cranial injuries included skull-base fractures, epidural hematomas, parenchymal hematomas, subarachnoid hemorrhages, and subdural hemorrhages. The values of each body region are given as percentages (and total numbers). The average of the affected body regions is given as total number (±standard deviation) and as affected portion, respectively. * = statistically significant difference.

	WBCT	WBMR	*p*-Value
Head	57.0% (89)	55.9% (75)	0.853
Face	28.8% (45)	11.9% (16)	<0.001 *
Thorax	33.3% (52)	19.4% (26)	0.008 *
Abdomen	17.9% (28)	35.8% (48)	0.001 *
Spine	18.5% (29)	29.1% (39)	0.035 *
Upper extremity	30.7% (48)	26.1% (35)	0.382
Lower extremity	6.4% (10)	24.6% (33)	<0.001 *
Vessels	3.8% (6)	0% (0)	-
Average of affected body regions	2.4 (±1.2)	2.2 (±1.1)	0.146
Average of affected body regions in relation to all body regions	29.4% (46)	26.9% (36)	0.146

**Table 7:** Abbreviated Injury Scale (AIS) in relation to the particular body region is presented as a mean AIS value (±standard deviation). Summarized AIS ≤ 2 values are expressed as percentages (and total numbers). * = statistically significant difference.

AIS	WBCT	WBMR	*p*-Value
Head	2.2 (±0.9)	2.0 (±0.6)	0.061
Face	1.3 (±0.6)	1.1 (±0.3)	0.003 *
Thorax	2.2 (±1.1)	1.2 (±0.5)	<0.001 *
Abdomen	2.6 (±1.5)	1.2 (±0.5)	<0.001 *
Spine	2.1 (±1.2)	1.5 (±0.6)	<0.001 *
Upper extremity	1.7 (±0.8)	1.4 (±0.5)	<0.001 *
Lower extremity	2.3 (±1.0)	1.5 (±0.5)	<0.001 *
Vessels	2.2 (±1.3)	0 (±0)	<0.001 *
AIS ≤ 2	54.4% (85)	84.4% (113)	<0.001 *

**Table 8:** Data show the AIS ≥ 3 in relation to age and affected body regions. All values are given in percentages (and total numbers). * = statistically significant difference.

AIS ≥ 3 Related to Groups of Age	WBCT	WBMR	*p*-Value
0–6	7.0% (11)	6.7% (9)	0.911
7–12	10.2% (16)	8.9% (12)	0.708
13–16	39.1% (61)	1.4% (2)	<0.001 *
**AIS ≥ 3** **Related to Body Regions**			
Head	16.0% (25)	9.7% (13)	0.112
Face	1.2% (2)	0% (0)	-
Thorax	8.3% (13)	0.7% (1)	0.002*
Abdomen	9.6% (15)	0.7% (1)	0.001 *
Spine	3.2% (5)	2.2% (3)	0.729
Upper extremity	1.2% (2)	0% (0)	-
Lower extremity	12.8% (20)	2.9% (4)	0.002 *
Vessels	1.9% (3)	0.7% (1)	0.627

**Table 9:** Injury Severity Score (ISS) related to trauma mechanism for both groups. All values are presented as a mean ISS (±standard deviation). * = statistically significant difference.

	WBCT	WBMR	*p*-Value
ISS Severe trauma mechanism	9.5 (±7.7)	6.4 (±4.7)	0.001 *
ISS Slight trauma mechanism	7.2 (±5.8)	5.0 (±3.3)	0.048 *

**Table 10:** Hospitalization time in the intensive care unit (ICU) and overall time in the hospital are presented. All values are expressed as a mean value in days (±standard deviation). * = statistically significant difference.

	WBCT	WBMR	*p*-Value
Hospitalization in ICU in days	5.5 (±10.2)	0.6 (±1.6)	<0.001 *
Total hospitalization in days	15.0 (±32.8)	4.4 (±4.9)	<0.001 *

## Data Availability

The data presented in this study are available within the manuscript.
